# Early and stable difficulties of everyday executive functions predict autism symptoms and emotional/behavioral problems in preschool age children with autism: a 2-year longitudinal study

**DOI:** 10.3389/fpsyg.2023.1092164

**Published:** 2023-07-31

**Authors:** Elisabetta Lupi, Federico Tucci, Laura Casula, Roberta Lucia Novello, Silvia Guerrera, Stefano Vicari, Giovanni Valeri

**Affiliations:** ^1^Child and Adolescent Neuropsychiatry Unit, Bambino Gesù Children’s Hospital, IRCCS, Rome, Italy; ^2^Department of Physiology and Pharmacology, Sapienza University of Rome, Rome, Italy

**Keywords:** autism, everyday executive function, development, outcomes, longitudinal

## Abstract

**Introduction:**

Longitudinal studies of autistic children show that autism symptoms and emotional/behavioral problems vary and change over time. However, the factors that affect this variability remain far from certain and very little is known about what take place in the preschool period and the role of executive functions (EF).

**Methods:**

Here, we test the influence of stable difficulties in everyday executive functioning (EEF) during early childhood across 2 years on autistic symptoms and emotional and behavioral problems. Twenty-nine autistic children (24 males and 5 females) were assessed twice within the space of 2 years. At baseline (M = 29 months, SD =5.6 months), participants were assessed for EEF, cognitive development, autistic symptoms, and emotional/behavioral problems. At follow-up, we repeated the same assessment except for cognitive development.

**Results:**

The group with stable difficulties (across 2 years) in EEF during early childhood showed a worsening in the severity of autistic symptoms and emotional and behavioral problems compared with children without EEF difficulties (*p* < 0.05), and these effects cannot be attributable to cognitive development.

**Discussion:**

Our results suggest that early and stable EEF plays the role of a modifier by interacting with the core domains of autism, in particular with the social affect domain (SA CSS), influencing social cognition and exacerbating or lessening symptom expression and emotional behavioral problems. These short-term longitudinal and preliminary findings underscore the importance of EEF as necessary target for early intervention in children with autism.

## 1. Introduction

Autism spectrum disorder (ASD) is a neurodevelopmental condition characterized by deficits in social communication and interaction, hypo- or hyperreactivity to sensory stimuli, and restricted and repetitive behaviors and interests (American Psychiatric Association, 2013). Children and adolescents with ASD usually have impairments in executive functions (EF) which refer to cognitive constructs that encompasses a series of processes such as emotion regulation, coping strategies, attention maintenance, and management of flexible problem-solving (Pennington and Ozonoff, 1996; Sergeant et al., 2002; Hill, 2004; Kenworthy et al., 2008).

It is worthy of note that a recent meta-analysis confirmed broad executive dysfunction in participants with ASD and suggested that difficulties remain stable across development (Demetriou et al., 2018) or limited improvements can be observed over time (Anderson, 2002).

For example, no changes in concept formation ratings, impaired response inhibition over time, and lack of longitudinal improvement in working memory are commonly observed (Luna et al., 2007; Van den Bergh et al., 2014; Andersen et al., 2015b).

However, there is a lot of variability in EF study results and it is necessary to consider the type of tools used during assessment: neuropsychological or performance-based measures are reliable indicators of EF components (e.g., inhibition and working memory), but do not always represent everyday executive functions (EEFs) (Burgess et al., 2006; Gardiner et al., 2017) that refer to emotional and behavioral process such as inhibition, shifting, emotional control, working memory, planning, and organization observed by parents in everyday life settings (Gioia et al., 1996). In contrast to performance-based EF tasks, ratings of EEF represent real world difficulties of children during social interactions, shifting from one activity to another, or during new tasks (Kenworthy et al., 2008; Toplak et al., 2013). Moreover, it is within this perspective that EF difficulties are most evident (Rosenthal et al., 2013; Smithson et al., 2013; Blijd-Hoogewys et al., 2014; Granader et al., 2014; Van den Bergh et al., 2014), being more directly related to functional outcomes (Pugliese et al., 2016; Tsermentseli et al., 2018) and persistent across time (Rosenthal et al., 2013).

Furthermore, studies that focus on EF or EEF during the pre-school period are still limited (with participants below the age of 6 years) (Espy, 2004) and findings of EF/EEF impairments have revealed conflicting data (McEvoy et al., 1993; Dawson et al., 1998; Griffith et al., 1999; Dawson et al., 2002; Stahl and Pry, 2002; Yerys et al., 2007). For example, Valeri et al. (2020) found differences in EF performance in a group of very young children with ASD in shifting and inhibitory control compared with the control group (TD) and this was confirmed also by Garon et al. (2018). Conversely, previous studies (Griffith et al., 1999; Dawson et al., 2002; Yerys et al., 2007) found no specific EF deficit in preschool children compared with either control groups. This variability of results, in addition to other factors, could also be influenced by the difficulties in measuring EF and EEF within a condition, such as ASD, that is heterogeneous and often associated with a highly variable functioning profile and different developmental trajectories (Lord et al., 2020). Nevertheless, an association between EEF difficulties and other factors, including (a) the severity of autism symptoms (Leung et al., 2016; Torske et al., 2018), (b) adaptive functioning (Gilotty et al., 2002; Pugliese et al., 2016), and (c) emotional and behavioral problems (Wallace et al., 2016; Gardiner and Iarocci, 2018), as well as other problems (Rosello et al., 2018), was confirmed in children with ASD, but still few pieces of research have studied these relationships over time (Renty and Roeyers, 2006; Billstedt et al., 2011; Bishop-Fitzpatrick et al., 2016).

Considering the previous background and taking into account the potential source of variability of autism symptoms and emotional and behavior problems, it may be useful to understand the relationship between early and persistent difficulties in EEF and autism symptoms and emotional/behavioral problems in order to design appropriate intervention strategies.

### 1.1. The relationship between EF/EEF and ASD symptoms

The relationship between EF/EEF and autism symptoms in children and adolescents have been demonstrated with both performance-based and everyday report measures, but the results are still limited and contrasting (Liss et al., 2001; Ozonoff et al., 2004; Verte et al., 2006; Solomon et al., 2008; Kenworthy et al., 2009; Semrud-Clikeman et al., 2010).

The relationship between EF and restricted and repetitive behaviors (RRBs) of individuals with ASD has been well studied in school-age children and adults using performance-based tasks (Russell, 1997; Hill, 2004; Lopez et al., 2005; Ozonoff and Schetter, 2007; Yerys et al., 2009) and specific difficulties in inhibition and shifting have been hypothesized to have a relationship with RRBs (Turner, 1997). For example, Mosconi et al. (2009) showed that impaired inhibition of automated responses was related to enhanced higher order repetitive behaviors (e.g., compulsions) in people with ASD. South et al. (2007) found a link between RRBs and flexibility in a group of 19 individuals (ages 10–19) with high-functioning autism spectrum disorders (South et al., 2007).

Similarly Lopez and colleagues, when comparing adults with ASD and matched controls in an executive function battery (Delis-Kaplin Executive Function Scales; Swanson, 2005) found that several executive processes (i.e., cognitive flexibility, working memory, and response inhibition) were highly related to RRBs (Lopez et al., 2005). Also, D'Cruz and colleagues found that individuals with ASD had lower scores for flexibility tasks (reversal learning task) that correlated with the severity of RRBs (D'Cruz et al., 2013). Conversely, when considering preschool-age children (mean age = 2.9 years) with ASD, Yerys and colleagues found that no specific EF deficits were in relationship with autism symptoms compared with matched controls (Yerys et al., 2007). Regarding EEF, the links between the everyday rating of EF and RRBs have been found in 9–10 year old children: Kenworthy and colleagues, using the Behavior Rating Inventory of Executive Function (BRIEF) for assessment (Gioia et al., 2000), showed that everyday measures of inhibition, flexibility, and emotional control were related to the severity of RRBs (Kenworthy et al., 2009) and this relationship was partially confirmed by Semrud-Clikeman et al. (2010) in children with Asperger syndrome aged 9–16 years. Concerning the relationship between EF and social affect (SA) difficulties (i.e., social interaction, communication, and social cognition), different opinions exist and several authors have suggested that impaired EF have a relationship with social communication problems in individuals with ASD traits (Hill, 2004; Gökçen et al., 2016). Also, individual differences in theory of mind (ToM), which is frequently associated with EF, have been shown to predict children’s social interaction and communication difficulties (Tager-Flusberg, 2003). In a study conducted with preschool-aged children, a significant relationship was found between EF and social communication skills (McEvoy et al., 1993).

Supporting this, a recent longitudinal study highlighted the link between early EF deficits and later autism symptom severity (such as SA difficulties) after about 12 years (Kenny et al., 2019). When considering the ecological perspective, Gilotty et al. (2002) found that EEF components (initiation and working memory) had a relationship with social interaction and communication (Gilotty et al., 2002). Conversely, from a performance-based perspective, flexibility (a specific component of EF) has not been recently reported to be in relation with socio-communication abilities in children with ASD (D'Cruz et al., 2013; Reed et al., 2013). Also, Yerys et al. (2009), in a sample of school-age children, reported no correlation between a specific component of EF (set-shifting) and social or communicative symptoms in ASD. To our knowledge, there are very few studies investigating the link between EF and autism symptoms during the preschool age period: only Pellicano and colleagues showed that performance in an EF battery predicted later social cognition (Pellicano, 2007, 2010). Similar findings (Faja et al., 2016) showed that EF during the preschool period significantly predicted pre-symbolic and symbolic play skills (often associated with social cognition) at the age of 6 in a sample of children with ASD.

### 1.2. The relationship between EF/EEF and emotional and behavioral problems in ASD

EF play an important role in the mental health of children with ASD (Gardiner and Iarocci, 2018) and it has been estimated that the prevalence rate for anxiety is greater than 84% (White and Roberson-Nay, 2009) and 38% for depression (Magnuson and Constantino, 2011), with important implications on social skills and the ability to cope and adapt to the difficulties of everyday life (Kim et al., 2000; Johnston and Iarocci, 2017). In general, ASD is frequently associated with emotional and behavioral problems of both internalizing (e.g., mood disorders and anxiety) and externalizing difficulties (e.g., irritability, aggressiveness, and behavior difficulties) (Bauminger et al., 2010; Rosenberg et al., 2011; Strang et al., 2012). Although there are many diagnostic challenges in detecting them, it is necessary to take into account early predictors of emotional and behavioral symptoms (Riggs et al., 2006) and long-term studies with TD children suggest that attention should be paid to the development of EEFs in this process (Riggs et al., 2006; Martel et al., 2007; Vogan et al., 2018). However, studies examining the relationship between EF and/or EEF and emotional and behavioral problems in pre-school children with ASD are still scarce: some studies found relationships between EF difficulties and anxiety, depression, and aggressiveness in youths with ASD (Hollocks et al., 2014), but other studies failed to find relationships with performance-based tests (Simonoff et al., 2012; Andersen et al., 2015a,b). Interestingly, from the ecological perspective, Lawson et al. (2015) demonstrated a link between anxiety and depression that was mediated by EEF using the Behavior Rating Inventory of Executive Function assessment (BRIEF; shift scale; Gioia et al., 2000) in school-age children and adolescents with ASD, and Wallace et al. (2016) found that everyday measures of shifting had a relationship with anxiety among adults with ASD, whereas planning and organization abilities predicted depression. Partially in contrast with previous findings, Gardiner and Iarocci (2018) found that BRIEF (Gioia et al., 2000) index scores were unrelated to anxiety, but behavior regulation, a component of EEF, was significantly associated with depression symptoms for children with and without ASD.

### 1.3. Research aims

In summary, these findings provide some evidence that a possible factor contributing to the variability of autism symptoms and emotional/behavioral problems in children with ASD might be individual differences in EF and EEF. As an informant questionnaire-based approach is different and complementary to performance-based tasks (Meltzer and Krishnan, 2007; Kenworthy et al., 2008), our study focused on parent-reported EF measures using the Behavior Rating Inventory of Executive Function – Preschool Version (BRIEF-P) (Gioia et al., 1996), which takes into account everyday difficulties in the EF of preschool children. We examined the general EEF domain (GEC), in order to provide a broad view of everyday executive functioning. In fact, the EF structure reflects a collection of cognitive process (e.g., working memory, inhibition, and flexibility), but this appears to be inapplicable in early childhood (Wiebe et al., 2011). This is in line with Wiebe et al. (2008), who found that, in preschool-age children (3-6 years old), one EF factor best fit with the neurodevelopmental prospective. Recently, Hughes et al. (2009) also found that a global and unitary EF factor best represented preschool children’s performance in a two time point study where children were assessed at 4 and 6 years of age (Hughes et al., 2009). The present preliminary study aims to investigate whether early and stable EEF difficulties predict later autism symptoms and/or emotional/behavioral problems in children with ASD after 2 years of follow-up. A better knowledge of this relationship in preschool-age children with ASD could offer an insight into the complexity of the condition, which is extremely important for caregivers, teachers, and clinicians. Furthermore, an enhanced understanding of these links may allow for better assessment and the implementation of knowledge for specific targeted interventions.

## 2. Methods

### 2.1. Participants

The present study was conducted from January 2017 and December 2021 at the Child and Adolescent Neuropsychiatry Unit at the Bambino Gesù Children’s Hospital in Rome. The Ethical Committee of the Istituto Superiore di Sanità (Rome, Italy) approved the experimental protocol and methods (code: WFR- NET-2013-02355263) and informed consent was obtained from all study participants prior to the start of the present work. Ethical standards (Declaration of Helsinki) were applied.

The inclusion criteria to participate in the study were:

1. An age range between 24 and 36 months at baseline.
2. Two time points assessment across 2 years (including the assessment of EEF).
3. All participants underwent the same assessment and ASD diagnosis was confirmed by clinical observation conducted by child psychologists and psychiatrists using DSM-5 criteria (American Psychiatric Association, 2013) and confirmed with Autism Diagnostic Interview Revised (ADI-R) (Lord et al., 1994) and Autism Diagnostic Observation Schedule- Second Edition (ADOS 2) (Lord et al., 2012). All children scored above the cutoff score of ADI-R [(A) total cutoff = 10; (B) (V) total cutoff = 8; (B) (NV) total cutoff = 7; (C) total cutoff = 3; (D) total cutoff = 1] and ADOS-2 according to calibrated severity scores (CSS; Hus et al., 2014; Esler et al., 2015).
4. Absence of general medical, neurological, perinatal and genetic conditions.
5. All children participating in the study had undergone interventions based on applied behavior analysis with no specific focus on EFs over the 2 years.

An initial sample of 53 children with ASD was recruited, but only 45 children completed the two time point assessments. This sample was then stratified into three subgroups according to the GEC BRIEF-P at baseline (BL) and follow-up (FU):

– Children with normal scores (≤64) at BL and FU were classified as stable EEF+ (*N* = 19).
– Children with clinical scores (≥65) at BL and FU were classified as stable EEF (*N* = 10).
– Children with clinical scores and normal scores at BL and/or FU were classified as variable EEF (*N* = 16).

In view of the exploratory and preliminary nature of this study, we decided to conduct our analyses only on stable groups (EEF+ and EEF− stables) because, to focus on the influence of EEF, we considered it more useful to evaluate children with permanently working or permanently impaired EEF. Otherwise, the results obtained from data of children with variable EEF could be biased by contextual factors which can affect the results. Therefore, of the remaining sample of 45 participants, only 29 children with ASD were selected to carry on the principal analysis (Table 1).

**Table 1:** Demographical and principal characteristics of the sample (*N* = 29).

	Number	Gender (M/F)	Age in months (Mean ± SD)	GMDS-ER DQ Tot	ADOS 2 Tot	ADOS 2 CSS SA	ADOS 2 CSS RRB	CBCL TOT	CBCL EXT	CBCL INT
Total group	29	24/5	29 ± 5.6	69 ± 12.8	6 ± 1	7 ± 1	7 ± 1	59 ± 8	56 ± 10	60 ± 8

GMDS-ER DQ Tot, Griffiths Mental Development Scales-Extended Revised Developmental Quotient Total (GMDS-ER Luiz et al., 2006); ADOS-2 Tot, Autism Diagnostic Observation Schedule Second Edition Total Score (Lord et al., 2012); CSS SA, Social Affect Calibrated Severity Score (Esler et al., 2015); CSS RRB, Restricted and Repetitive Behavior Calibrated Severity Score; CBCL TOT, Child Behavior Checklist 1½ − 5 Total Score (Achenbach and Rescorla, 2000); CBCL EXT, Child Behavior Checklist 1½ − 5 Externalizing Behavior; CBCL INT, Behavior Child Behavior Checklist 1½ − 5 Internalizing Behavior.

Once our sample was selected, our specific aims were to:

1. Investigate if there were differences in severity of autistic symptoms both on SA and RRB between the two subgroups (EEF+ and EEF−) at BL and at FU.
2. Investigate if there were differences in emotional/behavioral problems between the two subgroups (EEF+ and EEF−) at BL and at FU.

We performed the Fischer test for gender and t-test for age to avoid any differences between the two groups for the demographical and clinical variables. The demographical data of the EEF+ and EEF− group are summarized in Table 2.

**Table 2:** Demographical information of EEF+ (*N* = 19) and EEF− (*N* = 10) groups at baseline.

	EEF+	EEF−	Statistical test
Number	19	10	
Gender (M/F)	15/4	9/1	Fischer test: n.s.
Age in months (Mean ± SD)	29 ± 6.21	29 ± 4.51	T-test: n.s.

EEF+, stable and normal scores on BRIEF-P on General Executive Component (GEC) (Gioia et al., 1996); EEF−, stable and clinical scores on BRIEF-P GEC. n.s means not significant.

### 2.2. Materials

#### 2.2.1. Everyday executive function assessment

The Behavior Rating Inventory of Executive Function - Preschool Version (BRIEF-P) was used to assess daily executive functioning. The BRIEF-P includes 63 items on a Likert scale of 1 = never, 2 = sometimes, and 3 = often. The scales are: Inhibition, Working Memory, Displacement, Emotional Control and Planning/Organization. These five scales are summarized into three indices, the Inhibitory Self-Control Index (ISCI), Flexibility Index (FI), and Emergent Metacognition Index (EMI), as well as a Global Executive Composite Score (GEC). In our experiment, the BRIEF-P was rated by only one parent; the same parent rated BL and FU. Scores were converted to standardized T scores (M = 50, SD = 10) with T scores ≥65 (+1.5 SD) defined as clinical problems in EEF and higher scores indicating more severe impairment in functioning (Gioia et al., 1996; Isquith et al., 2014).

#### 2.2.2. Cognitive development assessment

As a control analysis, we assessed cognitive development only at BL. For this purpose, we used the Griffiths Mental Development Scales-Extended Revised (GMDS-ER; Luiz et al., 2006), a psychomotor developmental assessment tool that includes five different subscales (Locomotor, Personal-Social, Hearing and Language, Oculo-manual Coordination, and Performance) and also provides an overall score for each subscale and for the overall scale, the Developmental Quotient (DQ).

#### 2.2.3. Autism symptoms assessment

To assess the severity of ASD symptoms, we used the Autism Diagnostic Observation Schedule Second Edition (ADOS-2) Module T, Module 1, and Module 2, a semi-structured assessment of communication, social interaction, and restricted and repetitive behaviors (Lord et al., 2012). Recently, Esler et al. (2015) published calibrated severity scores (CSS) that were obtained from the ADOS-2 scores also within two subdomains, Social Affect (SA) CSS, and Restricted and Repetitive Behavior (RRB). Because the CSS is less influenced by child characteristics (e.g., verbal level and age), it can be used to provide more reliable estimates of ASD symptom severity (Hus et al., 2014; Esler et al., 2015). The ADOS-2 was repeated after two years of follow-up to test the experimental hypothesis.

#### 2.2.4. Behavioral and emotional problem assessment

The Italian version of the Child Behavior Checklist (CBCL 1 ½ -5) (Achenbach and Rescorla, 2000) has been used for the assessment of behavioral and emotional problems. The CBCL 1 ½ -5 is one of the most widely used checklists. The CBCL provides seven syndromic scales, three summary scales, and five DSM-oriented scales (DOS). Clinically significant scores are a T score of 64 and above for the summary scales and a T score of 70 and above for the syndrome-oriented and DSM scales. Scores between 60 and 63 for the summary scales or between 65 and 69 for the syndrome- and DSM-oriented scales are borderline. Scores below 60 for the summary scales or 65 for the other scales are not considered clinically significant. To test the experimental hypothesis, the Child Behavior Checklist (CBCL 1.-5) was repeated after 2 years of follow-up.

### 2.3. Statistical approach

The statistical analyses were performed using the STATISTICA software, version 10.0 (StatSoft Inc.,).^1^

After the usage of the Shapiro–Wilk test to determine whether the distribution of individual scores in each test and subscales approximated Gaussian distributions, we decided to carry on all the analyses with non-parametric statistical tests. Specifically, the Wilcoxon test was used for within-groups analyses and the Mann–Whitney U test was used for between-groups analyses.

This decision was made since we had two small groups, so non-parametric analyses would be more reliable and appropriate. We then used the Grubbs test (arbitrary threshold of *p* < 0.01) to exclude the possible presence of outliers that may alter the results. The test confirmed the absence of outliers.

The primary analysis was performed on the Child Behavior Checklist (CBCL 1½ − 5) scores, whereas a secondary analysis was made in order to see if the EEF− group could also show worse scores on scales assessing the severity of autistic symptoms. Finally, we carried out a control analysis to exclude that any results were due to differences in the overall cognitive levels between the two groups.

Because the CBCL test has many subscales and thus many comparisons to be made, we corrected the *p* value (Bonferroni correction) for 30 statistical comparisons (*p* < 0.05/30 = 0.001 = *p* < 0.05 corrected) to give greater strength to the obtained results. For exploratory purposes, a statistical threshold of *p* < 0.05 uncorrected was also used. For the ADOS-2 and GMDS statistics, we used the uncorrected value of p as a reference.

## 3. Results

### 3.1. Cognitive development (GMDS-ER) analysis between EEF+ and EEF− groups at BL

The mean and standard deviation of the scores obtained at GMDS-ER by the two groups at the baseline condition are shown in Table A1 of the Appendix.

The Mann–Whitney test (*p* < 0.05) showed no statistically significant differences between the EEF+ and EEF− groups. This was a confirmation that the general results observed did not depend on a cognitive difference between the two groups.

### 3.2. Autism symptoms severity differences between EEF+ and EEF− groups in ADOS total, CSS SA, and CSS RRB

The Wilcoxon test (*p* < 0.05) showed no statistically significant differences in the ADOS-2 subscales in the within-groups comparison considering baseline and follow-up in the EEF+ and in the EEF− groups (Table A2 in the Appendix).

**Figure 1:**
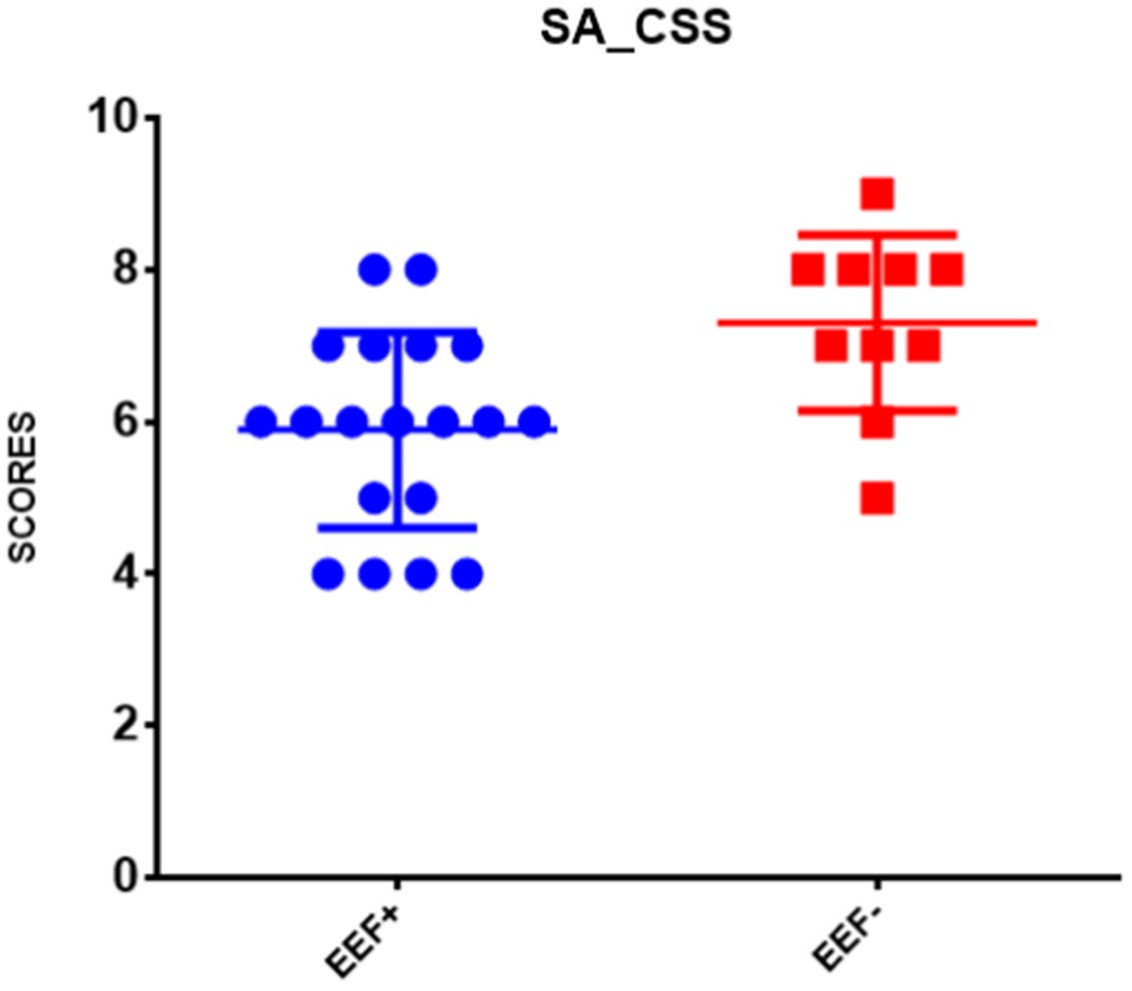
Comparison of the mean ± SD of the scores obtained in the ADOS-2 SA CSS at follow-up. Note: The ordinate axis refers to ADOS-2 SA CSS derived from Autism Diagnostic Observation Schedule Second Edition (Lord et al., 2012). EEF+, stable and normal scores on BRIEF-P on General Executive Component (GEC); EEF−, stable and clinical scores on BRIEF-P GEC (Gioia et al., 1996).

**Figure 2:**
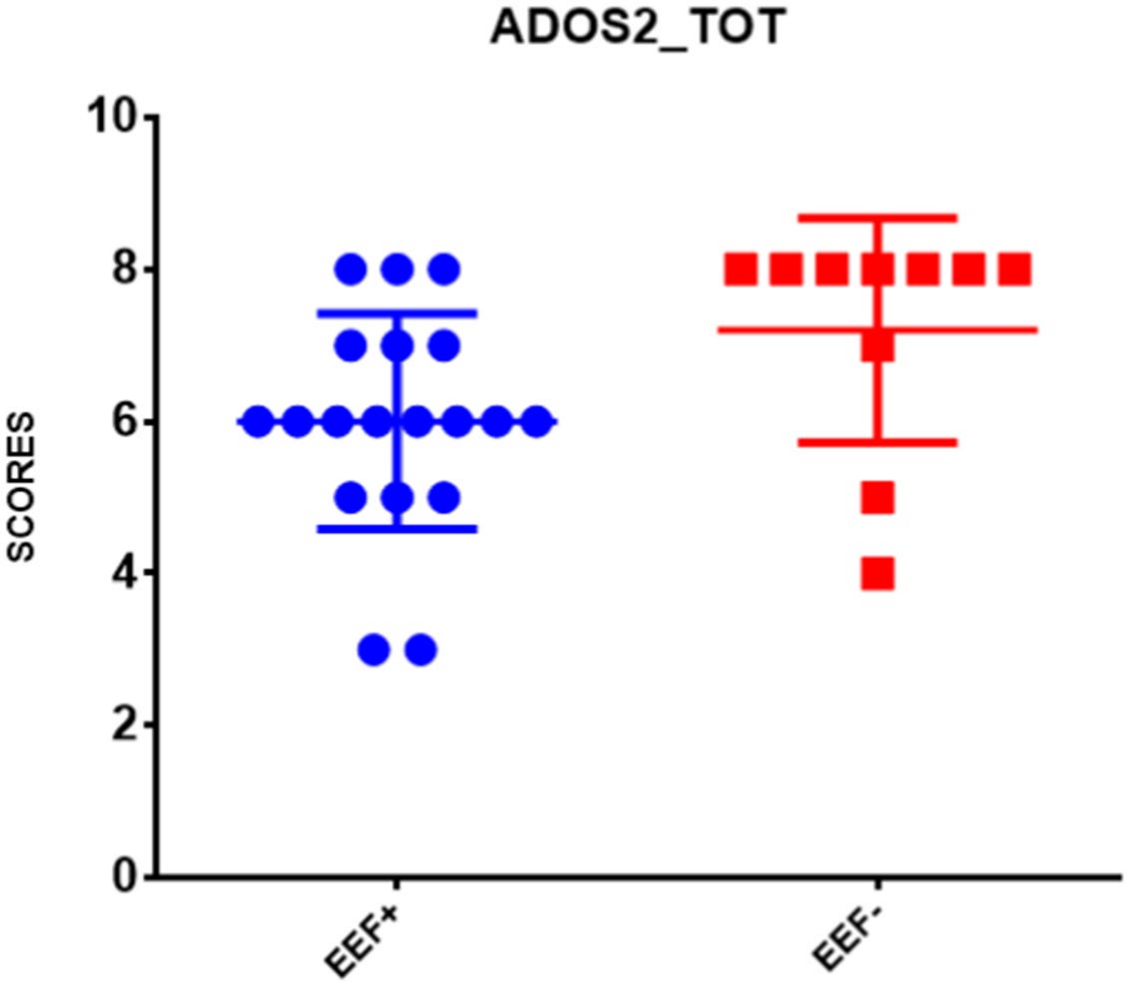
Comparison of the mean ± SD of the scores obtained in the ADOS-2 TOT CSS at follow-up. Note: The ordinate axis refers to ADOS-2 TOT CSS derived from Autism Diagnostic Observation Schedule Second Edition (Lord et al., 2012). EEF+, stable and normal scores on BRIEF-P on General Executive Component (GEC); EEF−, stable and clinical scores on BRIEF-P GEC (Gioia et al., 1996).

In the between-groups comparison, a statistically significant difference was found between the two EEF+ and EEF− groups with regard to the subscales ADOS-2 SA CSS and ADOS-2 TOT CSS measured at follow-up (Table A3 in the Appendix). Specifically, the EEF− group showed higher scores than the EEF+ group (Figures 1, 2).

### 3.3. Behavioral and emotional problems

Summarizing the CBCL within-groups results, we found that in the group of subjects with EEF+, there was a high improvement (lower scores) in the Withdrawn and Pervasive Developmental Prob subscales (Wilcoxon test, *p* < 0.05 corrected). Moreover, this improvement could also be observed in the Internalizing Problems, Externalizing Problems, Total Problems, and ADHD subscales (Wilcoxon test, *p* < 0.05; Table A4 in the Appendix). On the other hand, we observed higher scores obtained by the EEF− group in the Somatic Complaints subscale (Table A4 in the Appendix).

**Figure 3:**
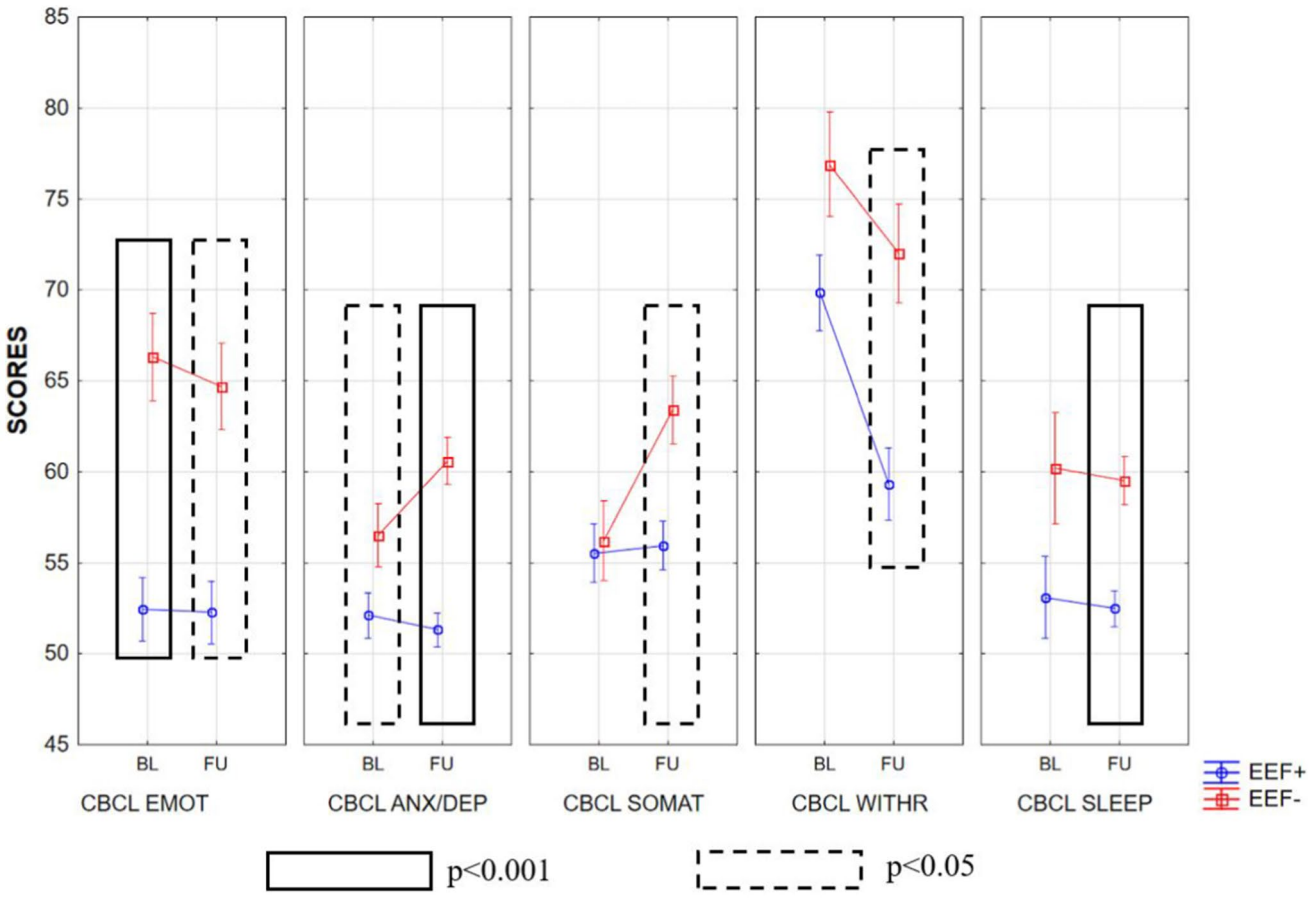
Mean ± SD of the scores obtained in the various subscales of CBCL at baseline and follow-up in the EEF+ and EEF− groups. CBCL EMOT, emotionally reactive; CBCL ANX/DEP, anxious/depressed; CBCL SOMAT, somatic complaints; CBCL WITHR, Withdrawn; CBCL SLEEP, sleep problems.

**Figure 4:**
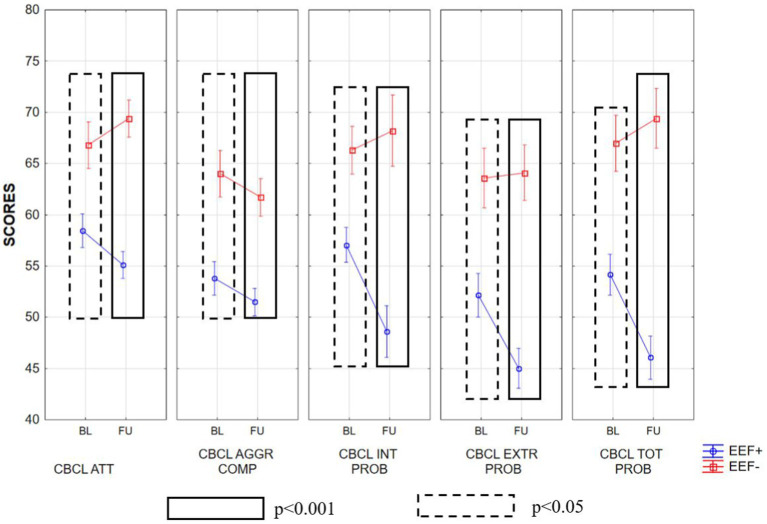
Mean ± SD of the scores obtained in the various subscales of CBCL at baseline and follow-up in the EEF+ and EEF− groups. ATT, attention problems; AGGR, aggressive behavior; COMP INT, internalizing problems; EXTR PROB, externalizing problems; TOT PROB, total problems.

**Figure 5:**
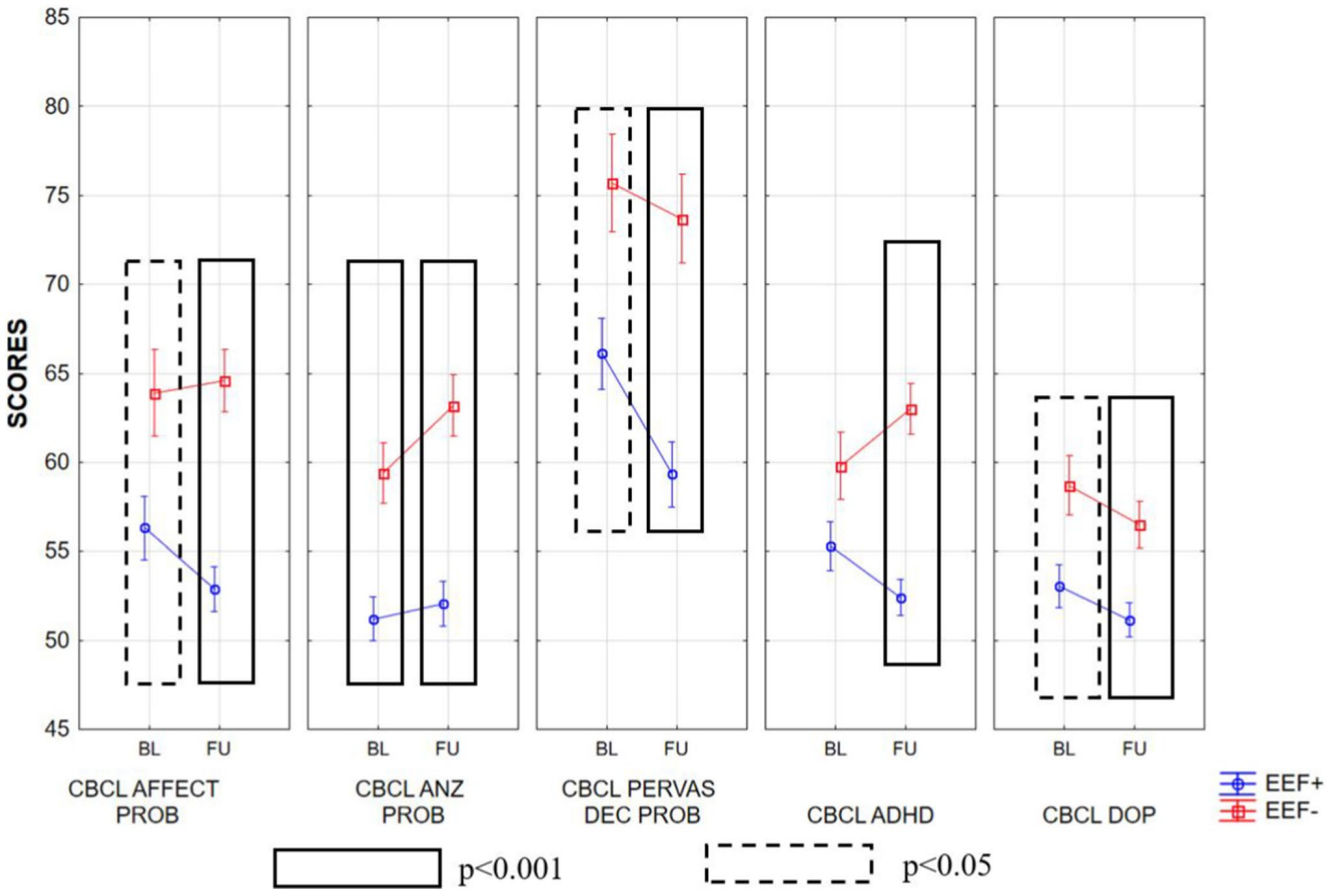
Mean ± SD of the scores obtained in the various subscales of CBCL at baseline and follow-up in the EEF+ and EEF− groups. AFFECT PROB, affective problems; ANX PROB, anxiety problems; PERVAS DEV PROB, pervasive developmental problems; ADHD DEP, oppositional defiant problems.

In the between groups comparisons, in the baseline condition, the EEF− group displayed significantly higher scores than EEF+ group in almost all subscales of the CBCL. When we looked at the scores obtained by children with ASD 2 years after baseline (follow-up), the EEF− group showed higher scores in almost all subscales, whereas the EEF+ group showed an overall improvement. These differences between the two groups were very pronounced, as shown in Table A5 in the Appendix; Figures 3–5.

The statistically significant differences observed between the two experimental groups increased when we considered follow-up. In fact, in general, the EEF+ showed lower and thus better scores in almost all subscales of the CBCL when compared to the EEF− group (Figures 3–5).

## 4. Discussion

Our preliminary results show that preschool-age children with stable difficulties in EEF across 2 years displayed an increase of mean scores in severity of autism symptoms compared with children without EEF difficulties. Furthermore, significant links between early EEF and behavioral/emotional problems have been demonstrated. In fact, our findings regarding autism symptoms demonstrated that stable difficulties in EEF seem to have a relationship especially with social affect (SA) difficulties, as previously indicated by others (McEvoy et al., 1993; Hill, 2004; Gökçen et al., 2016; Leung et al., 2016; Torske et al., 2018).

Moreover, data derived from some CBCL subscales in the within-groups comparison (CBCL within EEF+ BL vs. FU Withdraw subscale and Pervasive Developmental Problems subscale) give support to our findings regarding the severity of autism symptoms on ADOS-2 CSS SA and ADOS-2 CSS TOT. This result was further confirmed by the comparison between the two groups (CBCL between EEF+ vs. EEF) that showed that even in subscales where there was no difference to the BL (i.e., the Withdrawn subscale), the EEF+ group had a significant improvement at FU. Together, these results suggest that early and stable EEF plays the role of a modifier by interacting with the core domains of ASD, in particular with the social affect domain (SA CSS), influencing social cognition and exacerbating or lessening symptom expression and emotional behavioral problems (Mundy et al., 2007; Bedford et al., 2019).

This result is partially in line with studies that found that social cognition difficulties, resulting from EF, were related to social-communication symptoms in adolescents with ASD (Jones et al., 2018), in school-age children (Joseph and Tager-Flusberg, 2004), and in very young children (McEvoy et al., 1993). This association may emphasize the fact that many aspects of EF, in particular EEF, are important during sociality, during the initiation of social approach, in flexibility of social response, and in facial expressiveness during social interactions and self-regulation. However, in contrast with other previous studies (Turner, 1997; Lopez et al., 2005; South et al., 2007; Kenworthy et al., 2009), we did not find relationships between EEF and restricted and repetitive behaviors (RRBs), even though the lack of this relationship is consistent with prior results in the preschool-age range (Dawson et al., 1998). This data, if confirmed, would reinforce the need to study EEF from a developmental perspective, especially during the preschool-age period. Therefore, it is possible that some associations between EEF and RRBs during adolescence and adulthood are not present in preschool age children as previously shown by a recent longitudinal study in which RRB was evaluated with the Autism Diagnostic Interview-Revised (ADI-R; Lord et al., 1994) that suggested different developmental paths within this symptom domain (Richler et al., 2010).

We also found a relationship between stable EEF and emotional and behavioral problems. In particular, it has emerged that good and stable performance in EEF is associated with significant improvement over time in many aspects of emotional behavior problems (i.e., CBCL 1½–5 INT, EXT, and TOT). This result was partially confirmed by Gardiner and Iarocci (2018) and by Wallace et al. (2016) in a sample of older children with ASD. Our result is also in line with a recent study in school-age children that suggests lower emotion regulation, defined as a necessary behavior to deal with external standards (Bridgett et al., 2015), was a preventative factor for internalizing behavioral problems in children with ASD (De Lucia et al., 2021). A possible explanation for this phenomenon was suggested by Eysenck et al. (2007), in which anxiety interacts bidirectionally between the top-down attentional control system and the bottom-up stimulus-driven attentional systems (Corbetta and Shulman, 2002), reducing control over attentional allocation and contributing to the risk of developing internalizing symptoms.

Another finding concerns the supposed role played by EEF in preserving individuals from attention and hyperactivity problems after two years. In fact, children with adequate and stable EEF have fewer problems in attention and hyperactivity. Moreover, for similar attention-deficit and hyperactivity difficulties at BL, children with EEF− showed significantly worse scores on the CBCL ADHD subscale after 2 years. This is partially in line with Vogan and colleagues, who found that, in school-age children, prior estimates of EEF, measured with the BRIEF Emotion Regulation Index, predicted later externalizing behaviors (Vogan et al., 2018). Furthermore, the result concerning sleep problems (CBLC Sleep subscale) may also suggests that early and stable difficulties in EEF may have negative repercussions over time on the sleep quality of children with ASD. In fact, there is growing evidence of a relationship between poor sleep during childhood and EEF in ASD (Holingue et al., 2021; Tesfaye et al., 2021) though still few studies have investigated this relationship in the preschool-age period.

We also confirmed that, for some children with ASD, stable impairments of EEF persist for 2 years and this is partially in line with other previous studies (Geurts et al., 2009; Rosenthal et al., 2013; Smithson et al., 2013; Granader et al., 2014; Van den Bergh et al., 2014) suggesting there might be a ceiling on the extent to which such abilities can develop in children with autism (Griffith et al., 1999).

However, future studies may better investigate the development of EF during early childhood to clarify the importance of stability instead of variability in EEF over time and how this may contribute to the severity of autistic symptoms and emotional and behavioral difficulties.

As far as we know, there are three hypotheses proposed for the development of EF in individuals with ASD: the first hypothesis suggests that EF development in children and adolescents with ASD is delayed but follows a typical trajectory (Christ et al., 2011); a second hypothesis proposes a deviant EF development in ASD (Ozonoff and McEvoy, 1994); and the third hypothesis suggests a delayed but parallel EF development in childhood followed by a deviant EF development in adulthood (Luna et al., 2007). Our preliminary results add another possible perspective to the phenomenon: the importance of observing the tendency to improve (EEFs inc) or worsen (EEFs dec). It suggests that children with ASD and stable impairments of EEFs across two years during early childhood have the same developmental outcome regarding the severity of autistic symptoms and emotional behavioral problems as children with a decrease over time and vice versa (see Supplementary material). In fact, in the Supplementary material, we studied over time the two groups showing EEF variables—EEF increase “EEFinc” (with an improvement in EEFs over time) and EEF decrease “EEFdec” (with a worsening of EEFs over time)—that we did not consider in the main analyses. Preliminary and exploratory results showed that the group with EEFinc behaved like the EEF+ group while the EEFdec group was similar to the EEF− group. However, it would be appropriate for future studies to investigate this phenomenon on a larger sample to better understand the role of EEF in children with ASD.

Future studies will better investigate whether the tendency is more important than early observation at the single moment during the development process.

The current findings seem also to strengthen an independence between cognitive development assessed at the BL and EEF performance. This appears to be confirmed by the total score (GMDS-ER TOTAL DQ) and all other GMDS-ER DQ subscales. This independence, in addition to being an indicator of methodological quality, is also the confirmation of an already known result (Valeri et al., 2020) in a sample of preschoolers with ASD assessed with an Italian neuropsychological battery for preschoolers based on a functional perspective of cognitive domains (BAFE; Valeri et al., 2015).

In conclusion, since early EF difficulties appear to have significative repercussions after only 2 years on the severity of autistic symptomatology and emotional and behavioral problems, our findings may also contribute to supporting research on EF as possible endophenotypes for ASD following the Research Domain Criteria (RDoC) framework (Insel, 2014) which reflects the key characteristics of neurodevelopment (developmental trajectories/sensitive periods; Casey et al., 2014; Mittal and Wakschlag, 2017) and can be particularly relevant to the study of neurodevelopmental conditions.

Finally, early identification of EEF difficulties in children with ASD raises awareness of the need for targeted EF interventions (Diamond et al., 2007) during the pre-school period (Valeri et al., 2020) and underlines their relevance for social skills (Kenworthy et al., 2014) and emotional and behavioral problems over time (Hollocks et al., 2014; Lawson et al., 2015).

### 4.1. Limitations

The current study has some limitations. Due to the exploratory nature of the study, our sample was limited (29 children with ASD) and did not provide a comparison with a control group of TD or children with other neurodevelopment disorders. Moreover, the sample comes from a recruitment carried out on a clinical population and could be not representative of the general population. Finally, we decided to assess EF with parent report questionnaires, and this may be biased by the subjectivity of caregivers and a broad spectrum of other abilities of their children (e.g., language, cognitive, and motor processing). However, according to Bernstein and Waber (2007), EF are interconnected within neural networks that develop in the experiential context. So, in conclusion, we believe that the possibility of studying EF from an ecological perspective is also to be considered an enriching element for understanding the complexity of functioning of ASD.

## Data availability statement

The raw data supporting the conclusions of this article will be made available by the authors, without undue reservation.

## Ethics statement

The studies involving human participants were reviewed and approved by Ethical Committee of Istituto Superiore di Sanità (Rome, Italy) approved all the parts of the experimental protocol and methods described in this paper (code: WFR- NET-2013-02355263). Written informed consent to participate in this study was provided by the participants' legal guardian/next of kin.

## Author contributions

EL: conceptualization, formal analysis, investigation, and writing – original draft preparation. GV: conceptualization and investigation. FT: methodology and formal analysis. SG, LC, RLN, and SV: supervision and writing – review and editing. All authors contributed to the article and approved the submitted version.

## Funding

This work was also supported by the Italian Ministry of Health with “Current Research” funds.

## Conflict of interest

The authors declare that the research was conducted in the absence of any commercial or financial relationships that could be construed as a potential conflict of interest.

## Publisher’s note

All claims expressed in this article are solely those of the authors and do not necessarily represent those of their affiliated organizations, or those of the publisher, the editors and the reviewers. Any product that may be evaluated in this article, or claim that may be made by its manufacturer, is not guaranteed or endorsed by the publisher.

## Footnotes

^1^ www.statsoft.com
