# Supplementary material for: Early and stable difficulties of everyday executive functions predict autism symptoms and emotional/behavioral problems in preschool age children with autism: a 2-year longitudinal study
Source: Front Psychol. 2023 Jul 31;14:1092164. doi: 10.3389/fpsyg.2023.1092164 (PMC10425204; doi:10.3389/fpsyg.2023.1092164)
Supplement: Supplementary file 1 [file Data_Sheet_1.docx]

# 1 Supplementary Materials

2

1. Early and Stable Difficulties of Everyday Executive Functions Predict Autism Symptoms and
2. Emotional/behavioral Problems in Preschool age Children with Autism: a 2-Year Longitudinal Study 5

17

18 *Supplementary Methods*

19

1. We labelled children who improved as EEF increase (EEFinc), while we considered children who
2. worsened in EEF as EEF decrease (EEFdec).
3. The demographics characteristics of the 4 groups are summarized in Supplementary Table 1. 23
4. Table S1. Demographical information of EEF+, EEF-, EEFinc and EEFdec groups at Baseline.

|  | EEF+ | EEF- | EEFinc | EEFdec |
| --- | --- | --- | --- | --- |
| Number | 19 | 10 | 7 | 9 |
| Gender (M/F) | 15/4 | 9/1 | 7/0 | 6/3 |
| Age in months (Mean ± SD) | 29 ± 6.21 | 29 ± 4.51 | 30 ± 2.50 | 32 ± 4.20 |

1. Note: EEF+= stable and normal scores on BRIEF-P on General Executive Component (GEC) (Gioia et al. 1996); EEF-= stable and clinical scores on
2. BRIEF-P GEC; EEFinc = children with clinical scores on BRIEF-P GEC at Baseline but normal scores at Follow up; EEFdec = children with normal
3. scores on BRIEF-P GEC at Baseline but clinical scores at Follow up.

28

1. *Assessment and Statistical approach*
2. For the assessment we used the same methodology as in the main manuscript and we performed the
3. statistical analyses using the STATISTICA software, version 10.0 (StatSoft Inc., www.statsoft.com).
4. Statistical analyses were conducted using the Kruskal-Wallis test, a non-parametric correspondent of
5. the analysis of variance. We again used the Grubbs test (arbitrary threshold of p < 0.01) to exclude
6. the possible presence of outliers that may alter the result. The test confirmed the absence of outliers.
7. More complete and comprehensive information on the other parts of the methodology can be found
8. in the main manuscript.
9. *Cognitive Development (GMDS-ER) analysis between EEF+, EEF-, EEFinc and EEFdec groups at*
10. *BL*
11. Kruskal-Wallis test showed no significant differences in GMDS-ER TOT and in GMDS-ER
12. PERFORMANCE (Table S2).
13. Table S2. Mean±SD obtained in two subscales of GMDS-ER at Baseline, in EEF+, EEF-, EEFdec and EEFinc groups.

42

| **GMDS –ER BASELINE** | **EEF+** | **EEF-** | **EEFdec** | **EEFinc** | **KW test** |
| --- | --- | --- | --- | --- | --- |
| GMDS-ER TOTAL | 71.37±3.11 | 64.40±3.35 | 67.66±17.08 | 66.99±9.10 | p=0.40 |
| GMDS-ER PERF | 85.68±7.11 | 72.6±7.11 | 80.33±30.47 | 73.00±18.05 | p=0.29 |

1. Note: GMDS-ER TOTAL = Griffiths Mental Development Scales-Extended Revised Developmental Quotient Total (GMDS-ER Luiz et al., 2006);
2. GMDS-ER PERF = Performance Developmental Quotient. EEF+= stable and normal scores on BRIEF-P on General Executive Component (GEC);
3. EEF-= stable and clinical scores on BRIEF-P GEC; EEFinc = children with clinical scores on BRIEF-P GEC at Baseline but normal scores at Follow
4. up; EEFdec = children with normal scores on BRIEF-P GEC at Baseline but clinical scores at Follow up.

47

1. *Autism symptoms severity differences between between EEF+, EEF-, EEFinc and EEFdec groups in*
2. *ADOS Total, CSS SA and CSS RRB*

50

1. In the within comparisons, Wilcoxon test showed a statistically significant increase in the EEF-,
2. EEFdec and EEFinc considering the scores in both the ADOS 2 SA CSS (p < 0.05) and ADOS 2 TOT
3. CSS (p < 0.01) subscales.
4. On the other hand, we have found no differences between the four groups using the Kruskal-Wallis
5. test at the Baseline. However, Kruskal-Wallis test showed a significant difference (p<0.05) in ADOS
6. 2 SA CSS (p<0.01) and in ADOS 2 TOT CSS (p<0.01) at the Follow up. In particular, the EEFdec
7. group displayed higher scores than the other three groups. 58

59

60
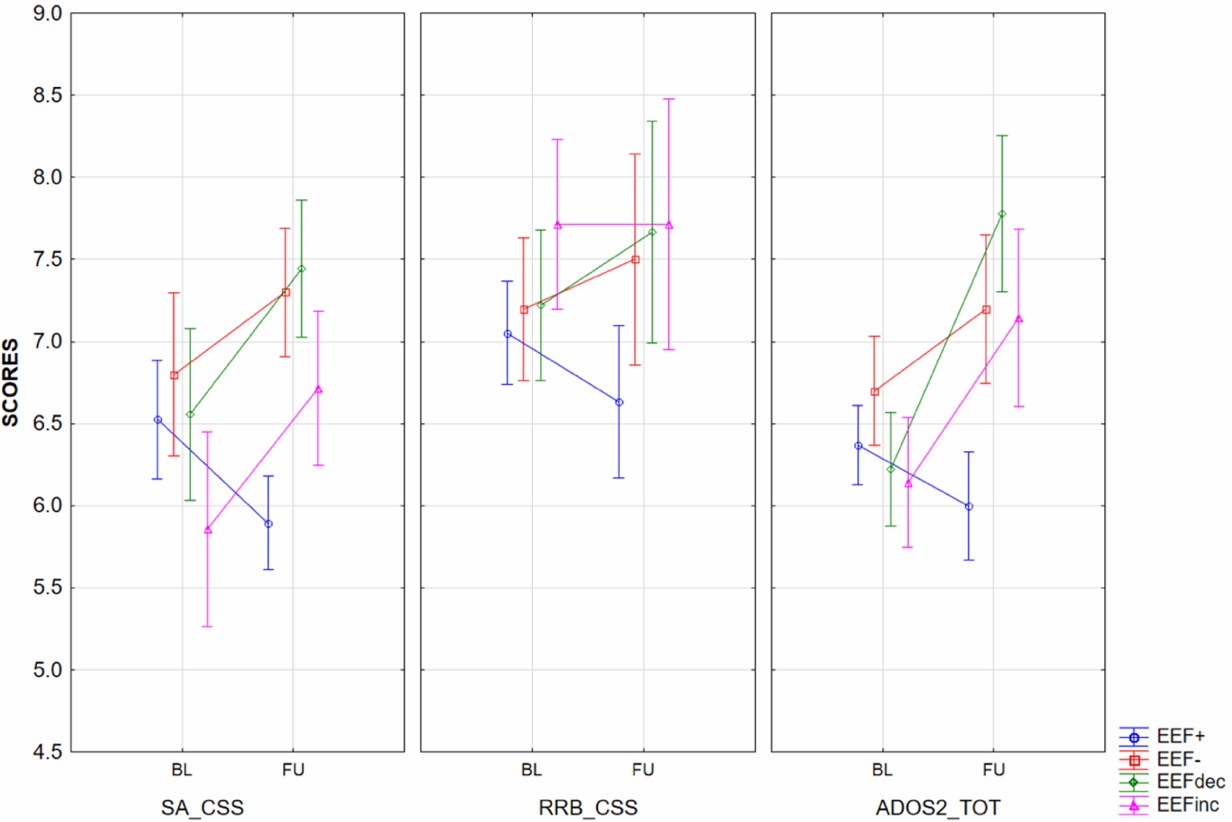


61

1. Figure S1. Comparison of the mean ± SD of the scores obtained in the ADOS 2 SA CSS; ADOS 2 RRB CSS and
2. ADOS 2 TOT CSS at the Baseline and Follow up. Note: Ordinate axis refers to ADOS 2 CSS at Autism Diagnostic
3. Observation Schedule Second Edition (Lord et al., 2012). EEF+= stable and normal scores on BRIEF-P on General
4. Executive Component (GEC); EEF-= stable and clinical scores on BRIEF-P GEC; EEFinc = children with clinical
5. scores on BRIEF-P GEC at Baseline but normal scores at Follow up; EEFdec = children with normal scores on
6. BRIEF-P GEC at Baseline but clinical scores at Follow up.

68

69 *Behavioral and Emotional Problems in the EEF+, EEF-, EEFinc and EEFdec groups*

70

1. The results in general between the four experimental groups show a very significant difference (p <
2. 0.01) in the EMOT, TOT PROB and ANX PROB subscales at the Baseline. As was also the case in
3. the main manuscript analyses, the differences between the four groups increase when Follow-up is
4. considered (Figure S2, S3, S4). Although the two groups were not included due to theoretical and
5. methodological problems in the main analyses, at the heuristic level, the EEFinc group tends to
6. behave similarly to the EEF+ group, while the EEFdec group tends to have score trajectories more
7. similar to those of the EEF- group. 78

79
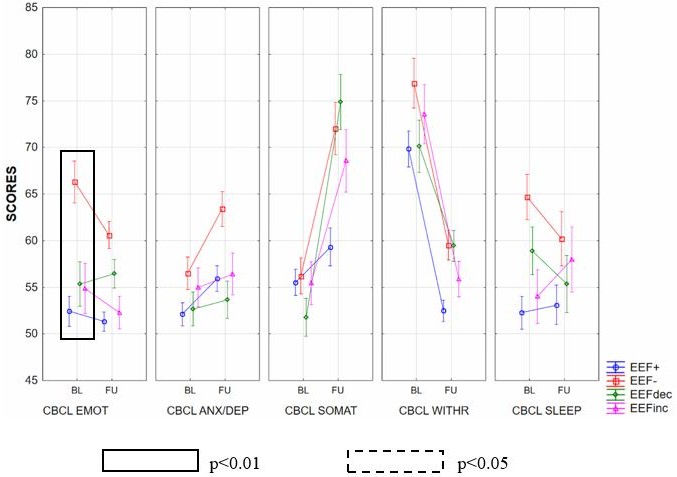


1. Figure S2. Mean±SD of the scores obtained in the various subscales of CBCL at Baseline and Follow up, in EEF+, EEF,
2. EEFinc, EEFdec groups. Note: CBCL EMOT= Emotionally Reactive; CBCL ANX/DEP=Anxious/Depressed CBCL
3. SOMAT= Somatic Complains; CBCL WITHR= Withdrawn CBCL SLEEP= Sleep Problems.

83

84


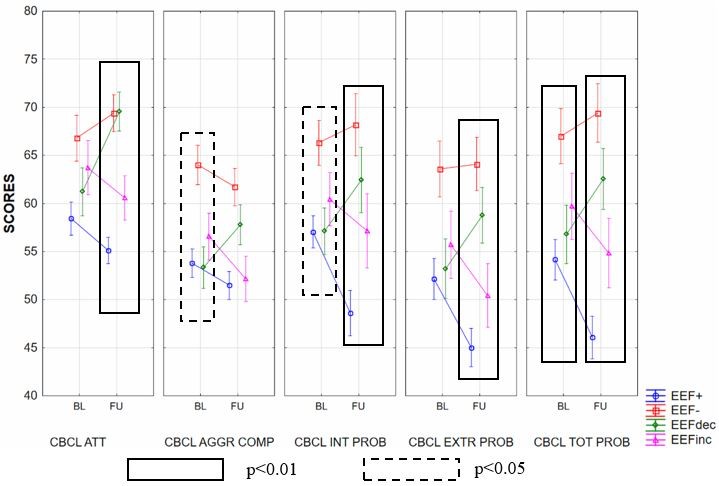
85

86

1. Figure S3. Mean±SD of the scores obtained in the various subscales of CBCL at Baseline and Follow up, in EEF+,
2. EEF, EEFinc, EEFdec groups. Note: CBCL ATT= Attention Problems; CBCL AGGR=Aggressive Behavior; CBCL
3. INT PROB = Internalizing Problems; CBCL EXTR PROB= Externalizing Problems; CBCL TOT PROB= Total
4. Problems

91

92

93

94
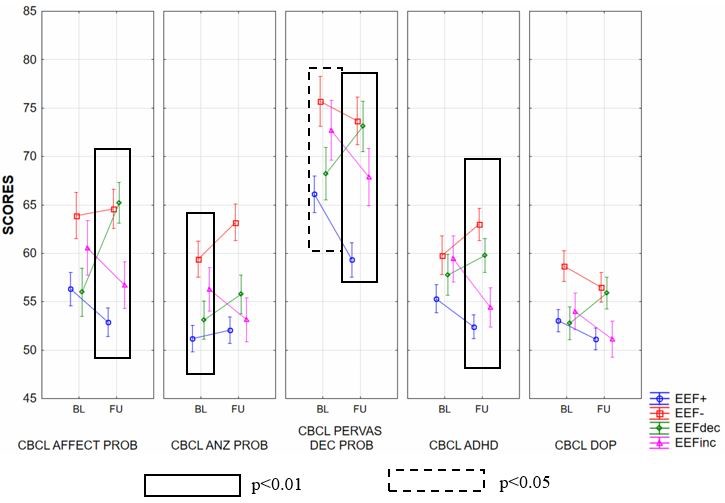


1. Figure S4. Mean±SD of the scores obtained in the various subscales of CBCL at Baseline and Follow up, EEF+, EEF-,
2. EEFinc, EEFdec groups. CBCL AFFECT PROB= Affective Problems; CBCL ANX PROB= Anxiety Problems; CBCL
3. PERVAS DEV PROB= Pervasive Developmental Problems; CBCL ADHD= Attention Deficit and Hyperactivity
4. Disorder; CBCL DOP= Oppositional Defiant Problems

99

1. *Correlation between Behavioral and Emotional Problems in the EEF+, EEF-, EEFinc and EEFdec*
2. *groups*
3. Moreover, we used the Spearman test (p < 0.05) for evaluating the correlation between the BRIEF P
4. and the CBCL tests. For this purpose, we calculated a global score for both tests by averaging the
5. various subscales for each individual subject. That correlation analysis was performed considering
6. all individuals as a whole group because the hypothesis was that Executive functions (BRIEF P) is
7. correlated with Behavioural and Emotional characteristics in ASD children in general. The result
8. (Figure S5) Spearman's correlation showed that there was a statistically significant (p < 0.01) and
9. positive correlation (r = 0.59) between the global BRIEF P score and the global CBCL score.
10.
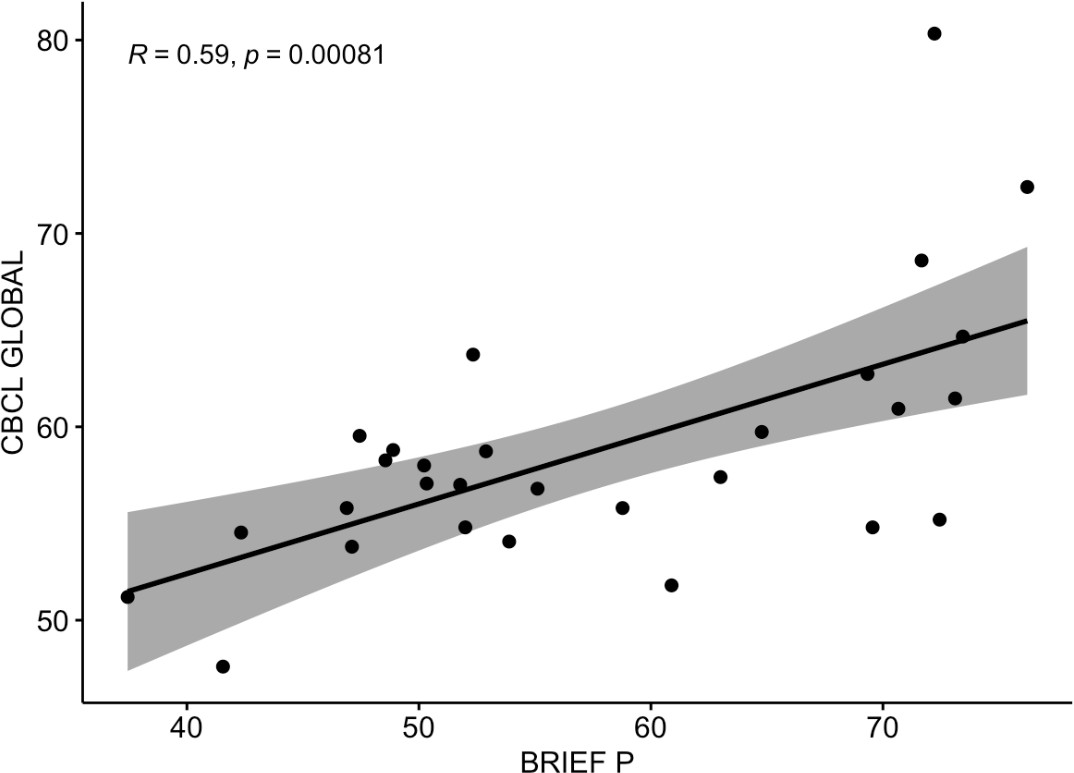
Specifically, the higher the scores at BRIEF P, the higher the scores at CBCL.

110

111 Figure S5. Scatterplot showing the (positive) correlation between the CBCL Global score and the BRIEF P global

112 score for the ASD children enrolled for the study (N=29).

113

114 *Supplementary description of the sample*

115

116 Since the sample is very small, we thought, in order to give a more precise and accurate description

117 of the data we used, to add a few descriptive tables showing median and interquartile values

118 (Supplementary Table S3, S4, S5). 119

120

121

122

123

124

125

126

127

128

129

130

131

132

133

134

Table S3: Descriptive values of EEF+ and EEF- groups for the GMDS scores.


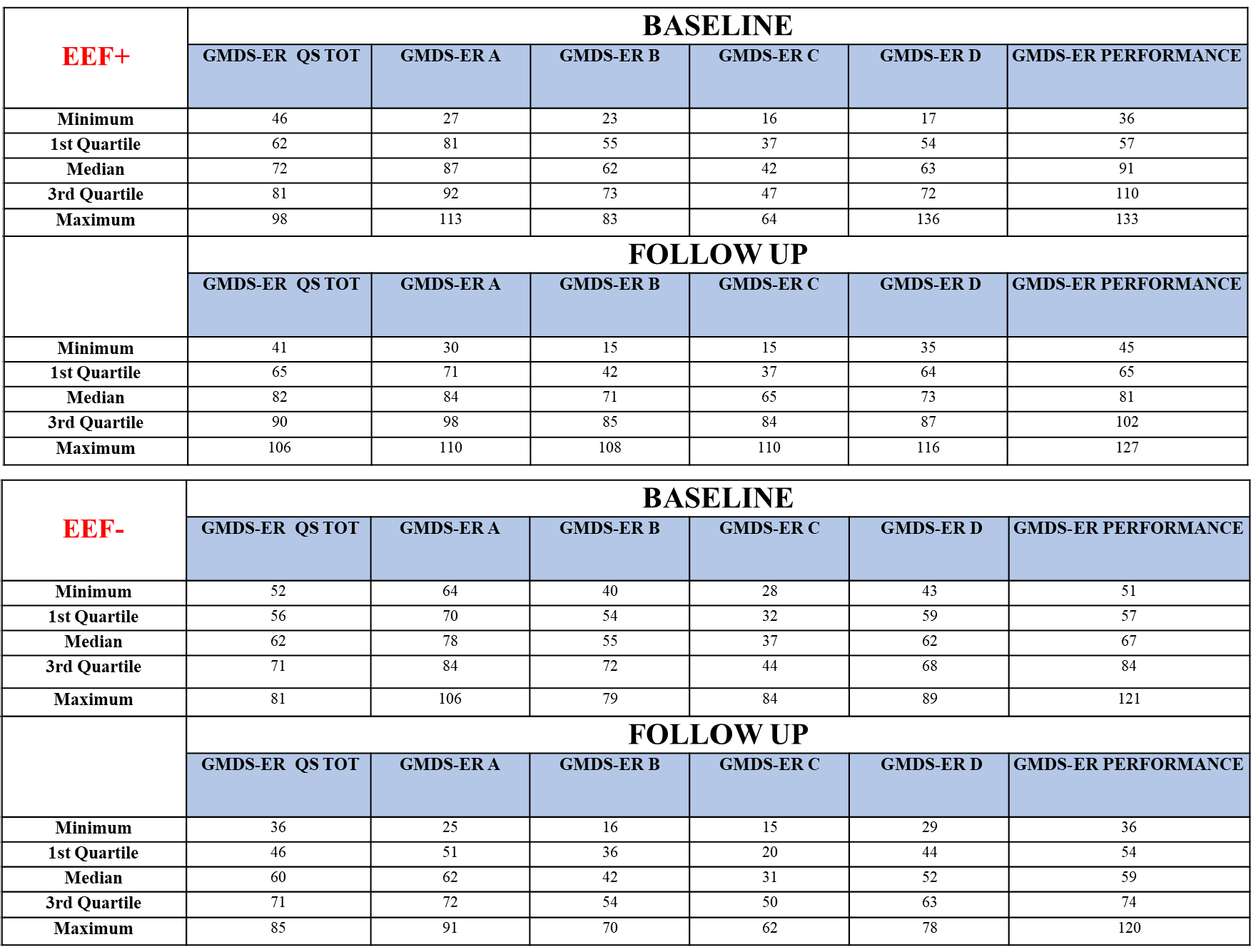


Table S4: Descriptive values of EEF+ and EEF- groups for the ADOS scores.


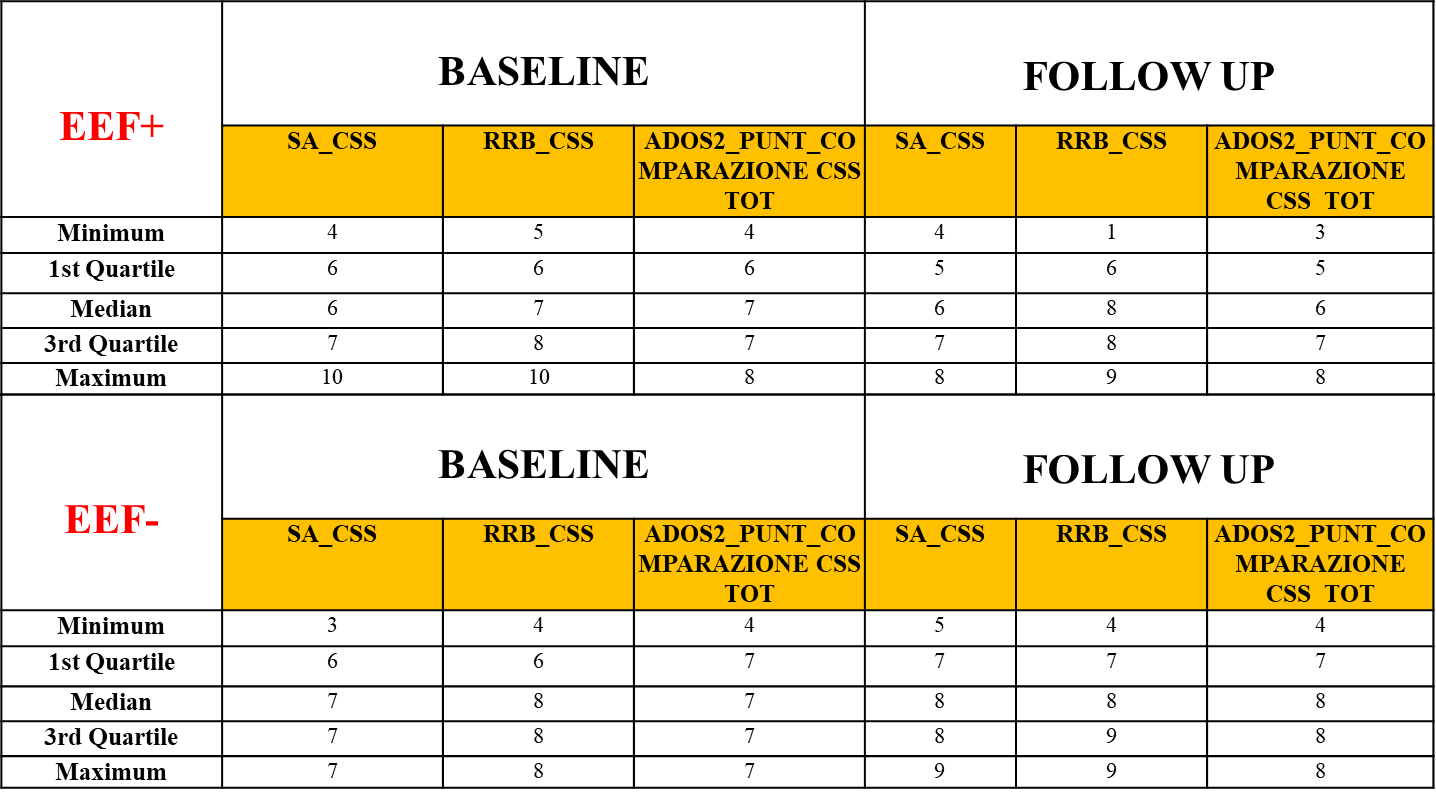


135 Table S5: Descriptive values of EEF+ and EEF- groups for the CBCL scores.

136
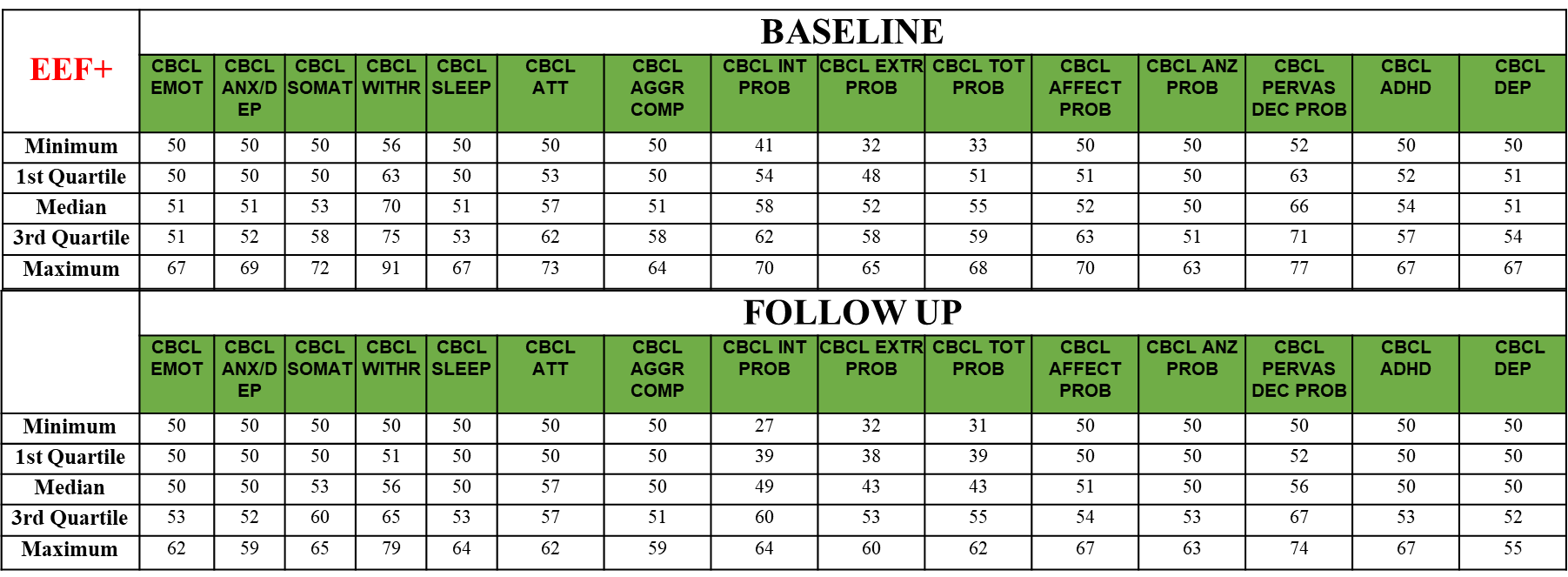


137
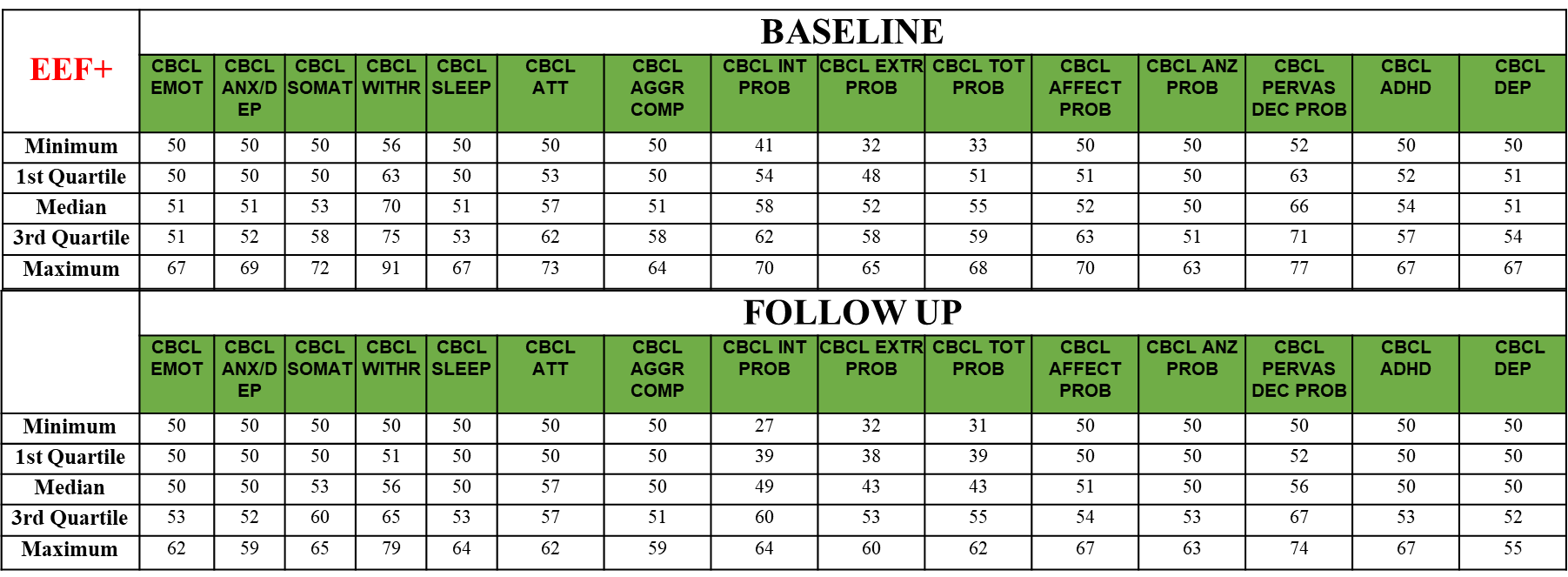


138

139

140

141

142
