# Supplementary material for: Early and stable difficulties of everyday executive functions predict autism symptoms and emotional/behavioral problems in preschool age children with autism: a 2-year longitudinal study
Source: Front Psychol. 2023 Jul 31;14:1092164. doi: 10.3389/fpsyg.2023.1092164 (PMC10425204; doi:10.3389/fpsyg.2023.1092164)
Supplement: Supplementary file 2 [file Data_Sheet_2.PDF]

Appendix: tables with all results

Table 1. Mean±SD and statistical test performed using T test Mann-Whitney test of the scores obtained in the various subscales of GMDS-ER at Baseline, in EEF+ and EEF-groups.

GMDS –ER BASELINE	EEF+	EEF-	Mann-Whitney test	Cohen's d
GMDS-ER TOTAL (DQ)	71.37±3.11	64.40±3.35	p = 0.13	0.57
GMDS-ER A (DQ)	85.05±4.21	79.1±4.13	p = 0.08	0.37
GMDS-ER B (DQ)	61.26±3.56	60.2±4.21	p = 0.58	0.07
GMDS-ER C (DQ)	41.53±2.51	41.9±5.14	p = 0.46	-0.03
GMDS-ER D (DQ)	64.32±5.56	63.8±3.94	p = 0.92	0.03
GMDS-ER PERFORMANCE (DQ)	85.68±7.11	72.6±7.11	p = 0.25	0.48

Note: GMDS-ER TOTAL DQ = Griffiths Mental Development Scales-Extended Revised Developmental Quotient Total (GMDS-ER Luiz et al., 2006); GMDS-ER A (DQ)= Locomotor Developmental Quotient, GMDS-ER B (DQ)= Personal-Social Developmental GMDS-ER C (DQ)= Hearing and Language Developmental Quotient; GMDS-ER D (DQ)= Oculo-manual Coordination Developmental Quotient; GMDS-ER PERFORMANCE (DQ)= Performance Developmental Quotient. EEF+= stable and normal scores on BRIEF-P GEC (Gioia et al. 1996); EEF-= stable and clinical scores on BRIEF-P GEC.

Table 2. Mean±SD and statistical test performed using Wilcoxon test of the scores obtained in the SA CSS, RRB CSS and Total score of ADOS-2 at Baseline and Follow up, in EEF+ and EEF- groups.

ADOS-2 (Within)				
GROUPS	TESTS	Baseline	Follow up	Wilcoxon test
EEF+	ADOS 2 SA CSS	6.53±0.35	5.89±0.30,	p=0.36
	ADOS 2 RRB CSS	7.05±0.32	6.63±0.54,	p=0.86
	ADOS 2 CSS TOT	6.37±0.23	6.00±0.32	p=0.35
EEF-	ADOS 2 SA CSS	6.80±0.59	7.30±0.37	p=0.25
	ADOS 2 RRB CSS	7.20±0.53	7.50±0.62	p=0.37
	ADOS 2 CSS TOT	6.70±0.33	7.20±0.47	p=0.37

Note: ADOS 2 Tot= Autism Diagnostic Observation Schedule Second Edition Total Score (Lord et al., 2012); CSS SA= Social Affect Calibrated Severity Score (Esler et al., 2015); CSS RRB = Restricted and Repetitive Behavior Calibrated Severity Score; EEF+= stable and normal scores on BRIEF-P on General Executive Component (GEC); EEF-= stable and clinical scores on BRIEF-P GEC (Gioia et al. 1996).

Table 3. Mean±SD and statistical test performed using Mann-Whitney test of the scores obtained in the SA CSS, RRB CSS and Total score of ADOS-2 Autism Diagnostic Observation Schedule Second Edition (Lord et al., 2012) at Baseline and Follow up, in EEF+ and EEF- groups.

ADOS-2 (Between)					
TIME	TESTS	EEF+	EEF-	Mann-Whitney test	Cohen's d
Baseline	ADOS 2 SA CSS	6.53±0.35	6.80±0.59	p=0.28	-0.17
	ADOS 2 RRB CSS	7.05±0.32	7.20±0.53	p=0.60	-0.10
	ADOS 2 CSS TOT	6.37±0.23	6.70±0.33	p=0.27	-0.33
Follow up	ADOS 2 SA CSS	5.89±0.30	7.30±0.37	p=0.003	-1.17
	ADOS 2 RRB CSS	6.63±0.54	7.50±0.62	p=0.16	-0.41
	ADOS 2 CSS TOT	6.00±0.32	7.20±0.47	p=0.01	-0.87

Note: ADOS 2 CSS TOT= Total Calibrated Severity Score (Esler et al., 2015); CSS SA= Social Affect Calibrated Severity Score; CSS RRB = Restricted and Repetitive Behavior Calibrated Severity Score; EEF+= stable and normal scores on BRIEF-P on General Executive Component (GEC); EEF-= stable and clinical scores on BRIEF-P GEC (Gioia et al. 1996).

Table 4. P values of the within statistical comparisons in EEF+ and EEF- groups.

CBCL (Within)		
Scales	EEF+ (BL) vs (FU)	EEF- (BL) vs (FU)
Emotionally Reactive	$p = 0.34$	$p = 0.07$
Anxious/Depressed	$p = 0.78$	$p = 0.13$
Somatic Complains	$p = 0.21$	**$p = 0.03$**
Withdrawn	**$p < 0.01$**	$p = 0.44$
Sleep Problems	$p = 0.63$	$p = 0.46$
Attention Problems	$p = 0.13$	$p = 0.44$
Aggressive Behavior	$p = 0.10$	$p = 0.54$
Internalizing Problems	**$p = 0.04$**	$p = 0.42$
Externalizing Problems	**$p = 0.01$**	$p = 0.56$
Total Problems	**$p = 0.01$**	$p = 0.47$
Affective Problems	$p = 0.08$	$p = 0.93$
Anxiety Problems	$p = 0.70$	$p = 0.19$
Pervasive Developmental Prob.	**$p < 0.01$**	$p = 0.70$
ADHD	**$p = 0.01$**	$p = 0.24$
Oppositional defiant problems	$p = 0.06$	$p = 0.46$

Note: Table 4 shows all the p-values, in bold red those significant for the Bonferroni correction ($p<0.05$) and in bold black those significant for the exploratory threshold ($p<0.05$ uncorrected).

Table 5. P values of the between statistical comparisons in EEF+ and EEF- groups.

Scales	EEF+ BL vs EEF- BL	EEF+FU vs EEF- FU
Emotionally Reactive	**$p < 0.01$**<br>Cohen's d = -1.89	**$p < 0.01$**<br>Cohen's d = -1.72
Anxious/Depressed	**$p = 0.03$**<br>Cohen's d = -0.83	**$p < 0.02$**<br>Cohen's d = -2.38
Somatic Complains	$p = 0.79$<br>Cohen's d = -0.10	**$p < 0.07$**<br>Cohen's d = -1.31
Withdrawn	$p = 0.06$<br>Cohen's d = -0.81	**$p < 0.02$**<br>Cohen's d = -1.53
Sleep Problems	$p = 0.83$<br>Cohen's d = -0.75	**$p < 0.05$**<br>Cohen's d = -1.74
Attention Problems	**$p < 0.08$**<br>Cohen's d = -1.22	**$p < 0.01$**<br>Cohen's d = -2.61
Aggressive Behavior	**$p < 0.02$**<br>Cohen's d = -1.47	**$p < 0.01$**<br>Cohen's d = -1.82
Internalizing Problems	**$p < 0.07$**<br>Cohen's d = -1.29	**$p < 0.01$**<br>Cohen's d = -1.86
Externalizing Problems	**$p = 0.01$**<br>Cohen's d = -1.54	**$p < 0.02$**<br>Cohen's d = -1.78

Total Problems	**$p < 0.02$**<br>Cohen's d = -1.02	**$p < 0.01$**<br>Cohen's d = -2.62
Affective Problems	**$p = 0.02$**<br>Cohen's d = -1.56	**$p < 0.01$**<br>Cohen's d = -2.20
Anxiety Problems	**$p < 0.03$**<br>Cohen's d = -1.15	**$p < 0.02$**<br>Cohen's d = -2.09
Pervasive Developmental Prob.	**$p = 0.02$**<br>Cohen's d = -0.78	**$p < 0.05$**<br>Cohen's d = -1.89
ADHD	$p = 0.08$<br>Cohen's d = -1.11	**$p < 0.01$**<br>Cohen's d = -2.43
Oppositional defiant problems	**$p = 0.03$**<br>Cohen's d = -1.11	**$p < 0.02$**<br>Cohen's d = -1.33

Note: Table 5 shows all the p-values, in bold red those significant for the Bonferroni correction ($p<0.05$) and in bold black those significant for the exploratory threshold ($p<0.05$ uncorrected).
